# Biological Functions of Rat Ultrasonic Vocalizations, Arousal Mechanisms, and Call Initiation

**DOI:** 10.3390/brainsci11050605

**Published:** 2021-05-09

**Authors:** Stefan M. Brudzynski

**Affiliations:** Department of Psychology, Brock University, St. Catharines, ON L2S 3A1, Canada; sbrudzynski@brocku.ca

**Keywords:** evolution of vocalization, ultrasonic vocalization, 22 kHz calls, 50 kHz calls, infant isolation calls, emotional arousal, mesolimbic dopaminergic system, mesolimbic cholinergic system, anxiety, hedonia, rat

## Abstract

This review summarizes all reported and suspected functions of ultrasonic vocalizations in infant and adult rats. The review leads to the conclusion that all types of ultrasonic vocalizations subserving all functions are vocal expressions of emotional arousal initiated by the activity of the reticular core of the brainstem. The emotional arousal is dichotomic in nature and is initiated by two opposite-in-function ascending reticular systems that are separate from the cognitive reticular activating system. The mesolimbic cholinergic system initiates the aversive state of anxiety with concomitant emission of 22 kHz calls, while the mesolimbic dopaminergic system initiates the appetitive state of hedonia with concomitant emission of 50 kHz vocalizations. These two mutually exclusive arousal systems prepare the animal for two different behavioral outcomes. The transition from broadband infant isolation calls to the well-structured adult types of vocalizations is explained, and the social importance of adult rat vocal communication is emphasized. The association of 22 kHz and 50 kHz vocalizations with aversive and appetitive states, respectively, was utilized in numerous quantitatively measured preclinical models of physiological, psychological, neurological, neuropsychiatric, and neurodevelopmental investigations. The present review should help in understanding and the interpretation of these models in biomedical research.

## 1. Introduction

Production of vocalization is one of the best means of communication in most terrestrial vertebrates, even though many physical conditions and environmental objects influence and impede sound transmission. Vocal communication is not dependent on daylight and visibility or on the proximity of organisms, does not leave permanent traces, and in most situations, is not critically influenced by air currents, humidity, or temperature. It is, thus, not surprising that the emission of vocalization for intraspecies communication is one of the oldest features present in vertebrates and tetrapods, ranging from lung fish to humans [1,2,3]. The neuronal mechanisms for the regulation of fundamental features of vocalization, such as call duration and sound frequency, are located in the deep hindbrain, bordering the spinal cord [1,4]. They are conserved in the vertebrate evolution and could be demonstrated in species ranging from toadfishes, such as midshipmen fish [5], to mammals, such as rats [6]. Sound frequency and duration of vocalizations are regulated by separate hindbrain nuclei [5], which allow for the generation of a large number of combinations of sound parameters, and thus, the generation of different signals with different information content that still use the same acoustic mode of communication.

The old phylogenetic history of vocalizations suggests that the vocal form of communication is highly adaptive and has been biologically important for animal behavior for hundreds of millions of years (approx. 400 million years for tetrapods) [7]. In this review, the emphasis will be on rat vocalization, which is the most extensively studied in rodents. In addition, some other mammalian species will be mentioned because only mammals have developed ventral myelinated vagal innervation, which originates from the nucleus ambiguous, innervates the larynx, and is critical for the generation of vocalization and the regulation of developed social interactions by the system termed social engagement system [8].

Studies of behavioral situations with the emission of ultrasonic vocalizations as indexes of emotional states have been extensively used as models of different neurodevelopmental, neurological, and psychiatric dysfunctions and diseases [9]. The magnitude and type of emitted ultrasonic calls were used in these models as a measure of the relevant effects. Therefore, there is a need for better understanding of the origin, nature, and role of rats’ ultrasonic vocalizations, their initiation mechanisms, their interpretation, and their equivalence to human vocal emissions.

Rat ultrasonic calls cannot be compared with human speech because speech is only a human function, and rodents do not have the necessary neural mechanisms and developed cognitive brain to have this way of communication. However, rat ultrasonic calls may be compared with human vocalizations. This is a valid comparison because human non-verbal vocalizations, such as crying, laughing, grunting, groaning, moaning, or shrieking, do not have lexical content, are generated by subcortical limbic mechanisms, and are evolutionary counterparts of other mammalian vocalizations (for classification of human verbal and non-verbal vocalizations as well as pathological vocalizations, see [10]). Although animal vocalizations have many subtypes and may convey referential information or be situation-specific (e.g., in species of prairie dogs [11,12,13]), they do not represent language in a human sense and do not have grammatical structure, sentences, words, syllables, or even fully translatable meaning. Naming mammalian vocalizations as syllables, particularly those emitted in series, is a misnomer and mistake that is still repeated in the literature. Animal calls (and human vocalizations) remain simple signals, even though they may have some specific situational content or may be emitted repeatedly or in a combination of calls. Vocalizations represent an evolutionarily older system of communication than human language with different neural regulation and different semiosis (“meaning”), and these two types of vocal communication should not be confused. Moreover, animal vocalizations were suggested to be interpreted as means of influencing the behavior of other individuals in a general way, rather than signals sending specific (e.g., lexical type) information to conspecifics as we know it from human language [14].

This review will attempt to facilitate understanding of functions of rat vocalizations, i.e., answering the question of why rats emit their calls. Different functions of vocalizations reflect our understanding of situations favoring vocal communication, and they do not mean that animals have many “understandings” or many intentional scenarios of call emission. Moreover, the emission of ultrasonic calls may serve more than one function at the same time. Thus, the classification of the functions of emitted vocalizations is used as a heuristic tool for the classification of the behavioral roles that vocalizations play in phylogenetic and ontogenetic history. Such a classification of documented or suspected functions of rat ultrasonic vocalizations has not yet been fully accomplished [15,16,17,18], and it will aid the ultimate goal of this review, which is to cumulate evidence supporting the hypothesis that all types of rat vocalizations, serving all biological functions, are driven by emotional arousal. Neural mechanisms that initiate emotional arousal, positive or negative, are, therefore, common in fulfilling any of these functions.

## 2. Evolution and Functions of Rat Vocalizations

### 2.1. Functions of Vocalization Originating from Mother-Infant Interactions

#### 2.1.1. Self-Preservation Function

The emission of vocalization evolved to serve many functions, but its primary and most important role was associated with saving the individual’s life and protecting the species since birth as we can observe today in altricial rat pups. The emission of infant vocalizations serve a self-preservation function, which is regulated by an innate, ancient emotional survival mechanism ascribed to the basic functions of the mammalian brainstem limbic system [19,20,21]. The communication of mothers with their offspring with vocalizations is regarded as the phylogenetically oldest form of vocal communication in rats and in all mammals [22]. The emission of calls by rat infants in the ultrasonic range was probably caused by increased air pressure in the respiratory system with constricted vocal folds that evolved in response to the cold [23,24], and this range of sound frequencies appears to have been highly adaptive. It may be speculated that, initially, the crying of infants and juveniles emerged and it was paralleled by the vocal responses of the mothers [22] since mostly females were primary caregivers to the offspring due to nursing and additional critical care (grooming, licking, nest attendance, etc.), without which rat infants could not survive.

In broader sense, maternal behavior was termed epimeletic behavior (from Greek *epimeleteon*—caregiving), which also includes paternal and biparental behavior [25]. On the other hand, pup or young vocalizations directed to their parents were termed et-epimeletic behavior (from Greek, *aeteo*—to beg, + epimeletic), and this label also includes signals other than vocalizations [25]. These terms were introduced at the beginning of the 20th century to make studies on behavior unbiased, devoid of colloquialisms, and easier to compare across species. While maternal vocalizations directed to pups were not carefully studied, etepimeletic vocalizations of pups focused the extensive attention of researchers.

#### 2.1.2. Locating Function

The emission of juvenile broadband vocalizations contains a primary locating function. The infant’s calls inform its mother as to where the pup is and help her to retrieve it back to her if it fell out of the nest, or to move it within the nest when the mother’s body compresses too strongly on the infant’s body [26]. The infant calls, termed sometimes as separation or isolation calls, or distress calls [27,28], provide mothers with critical information about the location of the vocalizing pup. Mothers orient toward the calling pup and approach it (phonotaxis) [29,30]. This maternal behavior is regulated not only by vocalizations but also by olfactory cues. In playback experiments, it was shown that mothers showed enhanced orientation toward the source of the infant’s calls if a silent pup was placed under a speaker [31]. Ultrasonic calls provide critical directional information for mothers, while pup odors determine the urgency and speed at which mothers begin searching [32,33].

Initial calls of pups after birth are not well exercised, and infants learn to emit the separation calls in such a way that maximizes the locating of the calling infant. In successive days after birth, the isolation calls gradually become longer and more complex, and the sound frequency within each call has a fluctuating character across many frequencies in a fashion similar to an ambulance siren [34], and they may reach significantly deep fluctuations. This pattern of emitting vocalizations could appear by the natural vocal selection that was demonstrated in infant rats [35]. Although there are some differences in the number of emitted calls, peak frequency, and frequency modulation among male and female pups of the main laboratory strains of Wistars, Long–Evans, and Sprague Dawley rats [36,37], the principle of locating the calling pup remains the same.

Pups of 14 days of age and older, however, will markedly reduce their emission of these calls when an adult male rat is nearby [38,39]. If a pup was isolated from the nest for a short time and retrieved, the mother will spend more time with it and pay more attention to that pup as compared to pups that have always been in the nest [40]. Maternal care is conserved in evolution and it can be demonstrated from rats to humans [41]. It has been shown that maternal care and proper vocal communication with the offspring contributes to development of the infants’ social brain and increases the offspring’s survival and their future reproductive success [42].

The importance of communication by vocalizations in rat infants may be further demonstrated by experiments showing that pups with less maternal help than usual were more anxious and emitted more infantile ultrasonic vocalizations than controls when they were separated from their mother and litter [43]. Additionally, experimental daily 30 min maltreatment of pups caused increased emission of their isolation calls as compared to pups receiving expected maternal care, and this procedure caused detectable epigenetic changes in the development of the brains of the maltreated rats [44]. Moreover, rats selected for higher emotionality traits emitted more ultrasonic calls when isolated as compared to pups with lower emotionality [45]. With the prolonged separation of pups from their mother, the acoustic parameters of their vocalization changed, and the pups emitted a larger proportion of high sound frequencies than the controls [46,47].

Pups pay constant attention to their mother and to her proximity. It was demonstrated that the interaction of pups with their mother just before the pup’s isolation further increased the pups’ vocalizations to a subsequent isolation (named maternal potentiation). If male rat contributed to caring for infants, pups would also show paternal potentiation [48,49]. It was concluded that infant separation vocalizations express infantile anxiety, and these vocalizations could be pharmacologically decreased by numerous anxiolytics [50,51].

#### 2.1.3. Protective Function of Maternal Care

The expression of infantile anxiety with the emission of calls also represents a protective function, which secures continued maternal help and safeguarding and builds a bond between the mother and her infants. Although many cues contribute to the development of the mother–infant bond, a repeatable emission of calls is one of the important signals. As studied in mice, infants at Postnatal Day 17 and 21 were able to recognize their own mother in a two-choice tests and preferred their own mother to a foreign mother [52]. In addition, based on the emitted calls, mouse mothers located their own pups faster than a stranger pup [52]. These bonds are the precursor of adult social bonds and are established by the release of oxytocin both in mice and rats [53]. The development of this bond is important at the infantile age. The creation of new stable bonds in adult rat social groups seems to be difficult or not possible, as it was studied in pairs of adult female rats [54].

The same protective function applies to the vocalization of human infants; it was suggested that excessive human infant crying may express anxiety of being abandoned, and crying prevents the withdrawal of parental help and secures continued care [55]. Loss of contact with the mother and the nest environment seems to be the primeval aversive emotional state expressed vocally [56]. Ensuring the continuation of mother–infant contact is one of the evolutionarily oldest and fundamental functions of emitting vocalizations.

### 2.2. Functions of Vocalization in Non-Agonistic Adult Social Interactions

#### 2.2.1. Phatic Communication Function

The mother–infant relationship developed another related function of vocalization, termed phatic communication, that is mostly characteristic of the rats’ adult life and seems to be appetitive. The term “phatic” was coined initially in anthropology as “bonding by language” [57], i.e., by emission of words (in humans) or vocalizations (in animals) that serve to create and maintain social bonds and closeness. The phatic communication in animals aims at maintaining connection between individuals, a reassuring proximal presence, and maintaining the cohesiveness of social groups in gregarious species [15]. The category of calls for establishing and maintaining contacts between adult members of the social group has been known for a long time. These vocalizations were later termed contact calls in classical ethological studies and were demonstrated in numerous species [58]. It has been even suggested that human humming may be regarded as the human equivalent of contact calls in social animals [59].

In rats, short-duration vocalizations, classified as flat 50 kHz calls, are used as contact calls and are emitted toward familiar conspecifics, even if these conspecifics are not present nearby [60,61,62]. Rats will particularly emit these calls when they detect fresh olfactory traces of other rats, and the more scent traces they detect, the more calls they emit, usually of a frequency-modulated type [63]. In the case of possible contact with many individuals, these calls may also play another, affiliative function (see below). It was also reported that rats may emit contact vocalizations in dyadic interactions or when being alone in a cage without detectable traces of other rats but shortly after separation from other companions [60,61].

Phatic communication has a character of the social announcement of presence, acceptance, and reassurance, and is not expected to be associated with approach. Phatic vocalizations may initiate similar reply calls from other rats but without further behavioral consequences. Such a mutual, infrequent calling is also evidence of social tolerance and potential social support, which would be associated with mutually positive emotional states. These calls may also have potential anxiolytic properties.

#### 2.2.2. Affiliative Function

Related to phatic communication is the emission of ultrasonic calls that may play an affiliative function. This function includes assuring non-agonistic, close interactions, a signaling approach, promoting direct contacts among individuals, causing grouping, and huddling. Unlike the phatic role of vocalizations, the affiliative function is associated with the approach and even direct contact among rats [64,65]. There is some recent evidence suggesting that 50 kHz calls are emitted during grooming [66] so they may contribute to anti-stress and to close-contact social behaviors driven by rat-positive emotional arousal. In a recent study summarizing results from selective breeding, devocalization experiments, and playback studies, a general, broad-term conclusion was reached that 50 kHz ultrasonic calls “serve as situation-dependent socio-affective signals with important communicative functions” [67].

The affiliative function of calls may be observed in many situations. Rats spend most of the daytime in underground burrows where vocal communication with ultrasonic calls is needed and particularly effective [68]. However, subterranean social mole-rats evolved communication with low-frequency vocalizations of 1.6–6.3 kHz [69]. The rat emission of affiliative calls has a calming effect on approaching conspecifics and prevents unexpected, aggressive attack. During a non-aggressive, ‘friendly’ approach [18], particularly in dark tunnels, the approaching rat will emit 50 kHz calls, usually in a characteristic short series of three frequency-modulated vocalizations emitted in rapid succession, presumably announcing its movement toward the other rat(s) (unpublished observations).

On the part of receivers, there is ample evidence that, when rats hear abundant 50 kHz calls, they approach the emitter or the source of the calls (e.g., a loudspeaker [65,70,71]. This effect is dependent on previous social experience [64], call-specific (no approach to 22 kHz calls), stronger in juveniles and females [67,70], and species-specific, thus other rodents, for instance, bank voles, do not show an approach response to the rat’s 50 kHz calls [72]. The perception of 50 kHz vocalizations and approach to them are clearly appetitive and rewarding responses to such an extent that rats can learn to self-administer the 50 kHz calls [73].

The extreme form of affiliative rat behavior is huddling. Huddling behavior, which is prevalent among infants, will also continue, to some extent, in adulthood [74,75]. While in infancy, one of the predominant roles of huddling is group thermoregulation [76], although other non-thermal stimuli are also important [77,78], adult huddling occurs less frequently and is associated with potential external danger or unfavored conditions, such as bright light or other dangers [79,80]. In these situations, rats have a tendency to crawl under other conspecifics, but this is not a blind behavior because males do not crawl under females but only under other males [81]. There are no studies recording the emission of vocalizations during this behavior.

#### 2.2.3. Passive Defensive Function

Ultrasonic vocalizations of infants, promoting huddling and nestling behavior, and calls serving affiliative function in juveniles and adults, that cause approaching and staying in close social groups have been jointly regarded as a form of “passive” defensive behavior of social groups and associations [82]. This basic defensive function is a form of primary defense, i.e., behavior without any detected presence of danger or predator (as distinguished from secondary defense, which appears when danger or a predator is present). This defensive behavior is a preventive form of behavior and is driven by an ancient limbic mechanism, aiming at securing potential social support and protection while being among other conspecifics. It is dubbed “strength and safety in numbers”, and it is present in all social mammals (and many other animal groups, e.g., fish and birds), including humans [83]. This defensive tendency must be a very old evolutionary development regulated by the oldest limbic mechanism.

#### 2.2.4. Socio-Coordinating Function

In general terms, phatic, affiliative, and related defensive functions of the emission of ultrasonic vocalizations are associated with a social, regulatory function not only at the juvenile level but in later social life, particularly in larger groups. This function was termed socio-coordinating function and particularly flat 50 kHz calls are involved [43]. Specific 50 kHz ultrasonic vocalizations are not one-to-one related to individual movements but are associated with specific patterns of motor behaviors and are suggested to coordinate moment-to-moment during social interactions among rats [84]. Experiments with the newly developed *Cacna1c* haploinsufficiency rat model demonstrated the importance of the socio-coordinating function, particularly during rough-and-tumble play behavior and during female interactions [85,86]. The deletion of *Cacna1c* in rats reduced the number of emitted 50 kHz calls, reduced social approach behavior during the playback of 50 kHz vocalizations, and revealed general deficits in communication and coordination during social behavior [85]. In other mammals, the coordinating role of calls is important in the initiation of movement of animal groups, which was well documented for white-faced capuchin monkeys [87,88].

#### 2.2.5. Social Buffering Function

It has been observed that the repeated tickling of rats (heterospecific play with a human hand) that was associated with the emission of vocalizations (mostly 50 kHz vocalizations) had a buffering effect on anxiety caused by handling or by the intraperitoneal injection of saline in these rats [89,90]. Although tactile stimulation during play and other cues are responsible for the buffering effect, it has been noticed that vocalization itself can also have a buffering function in other mammalian species [91]. It is conceivable that the emission of 50 kHz calls during grooming [66] may, jointly with tactile stimuli, have a buffering effect and may reduce stress and anxiety by the release of oxytocin. It was recently shown that juvenile and young rats that received repeated tactile stimulation with a human hand increased the emission of 50 kHz calls and showed the activation of oxytocin neurons in the hypothalamic paraventricular nucleus [92,93].

The abovementioned huddling behavior in rats also has an acute emotional buffering effect [80]. It is not known, however, what call types that rats emit in this situation, if any. However, the results with self-administration of 50 kHz calls by adult rats without any tactile stimulation may support the notion that the calls themselves could have a social buffering effect [73].

#### 2.2.6. Investigative Function

Rats are known to have well-developed responses to novelty, although not all individuals are high responders [94]. High responders to novelty have increased locomotor activity in the new environment and an enhanced level of dopaminergic activity in the nucleus accumbens, and the novel stimuli are rewarding for them [94,95]. In such novel situations, rat will emit vocalizations that play a positive, investigative role and are associated with rewarding novelty-seeking behavior and positive expectation. However, this response is dependent on the rat’s preliminary or pre-existing assessment of the environment or novel object. If the new environment seems to be rewarding, the rat will emit vocalizations, mostly 50 kHz calls. If, however, the new environment seems to be dangerous, the rat will not vocalize. This animal’s initial bias in its evaluation of the new environment or novel stimuli has been shown experimentally. Rats were initially trained to respond differently to acoustic stimuli as being positive or negative stimuli. Then, upon hearing the playback of 50 kHz calls or 22 kHz calls, the rats were presented with a new, ambiguous cue that was neither positive nor negative. The rats responded positively to the ambiguous cue when they heard 50 kHz calls, but responded to the same cue negatively when they heard 22 kHz calls [96].

In a recent experiment in a semi-novel environment, i.e., in a cage that had holes in the walls and was familiar to the rats but the lighting was changed so that the illumination of the cage had a novel element, rat ultrasonic vocalizations were recorded. Any time the experimental animal nose-poked the hole, the light was switched off for 5 s. The yoked control group was unable to switch the light off, but the on–off lights were controlled by the experimental group of animals [97]. It was found that rats that could control lighting performed more nose-pokes and emitted more vocalizations than the yoked rats. Long, alarm 22 kHz calls were not emitted; however, the experimental rats had significantly more nose-pokes with the emission of 50 kHz calls (with predominance of flat calls) and more nose-pokes with the emission of short 22 kHz calls (approx. 10–20 ms in duration) as compared to the control yoked group. Although the behavioral situation was not a typical novel environment, there was an element of novelty and novel exploration, and it was associated with calling [97]. It was concluded that 50 kHz calls and short 22 kHz calls could be associated with the investigative function and novelty seeking. The role of ultrasonic calls emitted by rats in novelty situation needs, however, more studies.

The role of call emission, particularly 50 kHz vocalizations, in novel situations could be interpreted as signaling a rewarding novelty-seeking behavior but it is situation dependent. This conclusion is supported by studies on mice tested in novel or familiar environments. Mice emitted significantly more high-frequency ultrasonic calls and of longer duration in dimly lit novel environments than in the brightly illuminated novel environment. In the bright-light conditions (aversive stimulus) of the novel environment, more calls were emitted with lower sound frequencies [98].

### 2.3. Functions of Vocalization in Social Play and Teamwork

#### 2.3.1. Ludic Function

The emission of vocalizations during social interactions within the nest evolved to perform another fundamental role during play behavior in juveniles and play-like behavior in adult rats [99]. The role of vocalizations aiding play is called ludic function or ludic behavior (from Latin *ludere*—to play), which is characteristic of immature animals [100]. The emission of 50 kHz calls is abundant during natural rough-and-tumble play (play fighting) in rats and occurs in anticipation of and during such play in juvenile rats [73,101,102]. The emission of 50 kHz vocalizations is associated with specific components of play behavior, and the calls function as play signals and signals maintaining playful mood and activity [99,103,104].

In general, the number of emitted 50 kHz calls was used as a quantitative index of the animal state both in juveniles and adults [15,105]. The number of emitted 50 kHz calls is not only the quantitative index of the magnitude of emotional arousal but the low levels of emission of these calls in infants and juveniles may inform about abnormal phenomena and may be indicative of potential prenatal damages to the limbic system as it was recently shown for prenatal exposure to valproic acid [106].

The emission of 50 kHz play calls can be also induced by a tickling procedure (heterospecific play with humans) that mimics natural rough-and-tumble play, and it has rewarding properties for rats [107,108,109]. The tickling procedure should follow two main events of rough-and-tumble play—dorsal contacts and pins separated by a short break—and should be repeated daily for the full effect [90]. The repeated tickling procedure can select groups of rats that will emit a particularly large number of 50 kHz vocalizations, signaling the appetitive value of the play [110]. Rats that refuse to play will emit low numbers of 50 kHz vocalizations, and even some 22 kHz calls, at the beginning of the play. Restrain stress applied to rats before tickling sessions significantly decreased the number of tickling-induced 50 kHz calls afterward [111].

Although adults rarely play, the appetitive value of light tactile stimulation, which only partially resembles rough-and-tumble play, still retained rewarding play value in young adults, and tactile stimulation induced emission of 50 kHz calls in habituated animals [60]. Interestingly, other forms of tactile stimulation by human hand in different parts of the rat’s body, which had low resemblance to natural rough-and-tumble play, or were entirely unnatural to rats (e.g., holding rats in a vertical position and touching their flanks), also induced the emission of 50 kHz calls, although at a lower rate than during natural play [60,112].

Tickling stimulation of adolescent and young adult rats is appetitive and has rewarding value, as it was demonstrated by the tickle-induced release of dopamine in the nucleus accumbens [113]. On the other hand, very light touching of the rats’ skin that failed to induce calling was not associated with the release of dopamine 113]. Therefore, tickling is not a purely tactile phenomenon but a procedure inducing a positive emotional arousal. A recent study has confirmed that the number of emitted 50 kHz vocalizations induced by tickling is proportional to the magnitude of positive emotional arousal and is, indeed, a good quantitative measure of this arousal as compared to other measures [114].

Another recent study reported that rats can play with humans in a “hide-and-seek” game. Rats not only learned how to play “hide” versus “seek” but they were emitting 50 kHz vocalizations during play with predominance of flat and frequency-modulated 50 kHz calls (approx. 72% of all emitted calls with 32% of frequency modulated calls) [115]. Since the authors were using a brief, abdominal tickling procedure any time the rat found a person, or was found by a person, a question arises as to whether rats really were playing “hide-and-seek” game in the human sense or had just learned some rules to merely try to get the tickle-like experience. Somewhat similar behavior was observed during the daily tickling procedure when the rats were escaping from the human hand that was trying to pin them and were approaching and chasing the human hand before dorsal contact [90,108].

The emission of 50 kHz calls during juvenile play fighting, which occurs more frequently in males, facilitates and maintains play behavior [99] that is important for male-typical brain development [116]. The emission of 50 kHz vocalizations contributes to maintaining play, regulates play, and indirectly serves as preparation for the young organisms to develop aptitude for general sociability (gregariousness). Play develops motor skills, even strengthens the skeletal apparatus, develops exploration skills, establishes social ranks and dominance, and prepares juveniles for aggressive and sexual behaviors with always present vocal communication [100,117]. As emphasized by Berlyne (1960) [118], who first proposed the term ludic behavior, play consists of a multitude of functional components, such as perceptual, cognitive, and motor activities, and emotional arousal [118]. Play is a vigorous and highly emotional positive behavior with a crucial role of vocalizations.

#### 2.3.2. Conative Function

The emission of 50 kHz vocalizations during ludic behavior also has another function, a conative function. This is an intentional action of an animal to catch the attention of one or more of the social group members and eventually influence their behavior in a general, non-specific way, or to mobilize them to common action/play. The emission of vocalizations subserving this function in young individuals may play just a general activation role but may change, e.g., into invitation to play. This function is well known in mammals, particularly observed in the vocalizations of domestic animals living with humans, and in vocalizations of attention-seeking human infants, where they are often interpreted as excessive crying for the “manipulation of parents” [55,119,120]. In rats, conative function of calling may be also well illustrated in infants as maternal/paternal potentiation. The vocalization of the isolated pup is significantly increased when the pup has been in contact with its mother immediately before isolation [48]. In this situation, calling cannot be directly associated with a lack of food or other stimuli, but aims at rapid maternal/paternal attention in a general way.

#### 2.3.3. Cooperative Function

Along with the development of the social life in rats, a new function of vocalization appeared that is termed cooperative function. An interesting experiment demonstrated this behavior in rats [121]. To receive a sucrose reward, pairs of familiar (to each other) rats were trained to simultaneously nose poke the holes to receive the reward. Cooperative behavior gradually increased over 44 days of training along with an increased emission of 50 kHz vocalizations. When the pair of rats was separated by a partition, blocking acoustic signals, the cooperative success deteriorated but reappeared again when the rats were separated only by a wire mesh partition and could hear each other. Thus, direct physical contact between the rats was not needed but the rats needed to communicate by ultrasonic calls to achieve cooperative success [121]. The emission of some ultrasonic calls was also suggested as a form of cooperation during common play actions in juvenile rats [122] and in sexual interactions [123].

### 2.4. Functions of Vocalization in Intraspecies Agonistic Interactions

#### 2.4.1. Agonistic Function

Adult rats use vocalizations for the regulation of their social life, e.g., for the establishment of dominance hierarchies, during aggressive encounters (mostly with intruders), during sexual behavior, feeding, defending territory, and in other situations requiring significant emotional arousal [75]. In all these situations, the emission of ultrasonic calls plays an important communicative role. Even an overall, rough analysis of all emitted vocalizations in these situations shows significant changes in many acoustic parameters of calls, suggesting that rats emit, at least, some calls specific for a given situation and behavior [124].

Aggressive behavior focused the most attention in research and emissions of 50 kHz and 22 kHz type of vocalizations were recorded during the aggressive attacks and defensive actions of the rats [125,126]. Aggressive/defensive behavior is associated with the highest emotional and autonomic arousal because it may be associated with significant body damage. The general label “aggressive behavior” includes many behavioral patterns, such as threatening, aggressive sideway posturing, directed attack, wrestling, boxing, kicking, punching, jumping, submissive posturing, chasing, and flight, so both offensive and defensive elements. Each of these elements is associated with a potentially different type and combination of emitted vocalizations, which may further differ among different rat strains [127]; however, detailed studies about the role of particular types of calls in these components of behaviors have not been systematically conducted.

The 50 kHz vocalizations are emitted mostly by the attacker and the 22 kHz calls by the defeated rat [127,128]. In the intruder–resident interactions, the intruder emitted mostly 50 kHz vocalizations that were changed to 22 kHz calls after its defeat [128,129]. Audible squeals are frequently emitted during fighting and intermingled with ultrasonic calls. Study of vocalizations emitted in the resident/intruder situation with a wire mesh preventing physical contact confirmed that mostly intruders emitted ultrasonic calls [130]. Interestingly, the ultrasonic calls emitted by the intruder were decreased by systemic morphine, but audible vocalizations were not sensitive to morphine, suggesting that their function is different from ultrasonic calls [130].

The emission of the ultrasonic vocalizations in these offensive/defensive situations plays an agonistic function (from Greek *agonisticos*—combative). This term was first suggested by Scott and Fredericson in 1951, mostly in relation to the complex behavior of rats and mice, and it comprises offensive and defensive groups of behaviors, including withdrawal, avoidance, and escape [25,131,132]. In addition to that, agonistic function includes elements of territorial behavior and defense against intruders.

Rats live in large groups that need space for nesting, hiding, and foraging, and will defend this space against rats from neighboring groups. It was postulated that one of the important functions of the evolution of adult vocalizations was spacing among neighboring animal groups. Vocalizations that can be received from a distance serve this purpose very well [133]. The territories rats defend are rather small and there is no good evidence that rats are defending large and defined boundaries around their living burrows [132]. However, agonistic behavior and relevant calling was shown to play broader functions in controlling population density, group stability, and partner choice [75]. Ultrasonic vocalizations used during territorial defense were not studied in rats, but in a study on mice, several types of calls were demonstrated, which were important in territorial defense [134].

#### 2.4.2. Appeasement Function

Many researchers have observed that the emission of long 22 kHz vocalizations during agonistic encounters by the defeated rat may have an appeasement function, which is widely observed in animal behavior [135]. The emission of these calls would decrease or inhibit further attacks of the aggressive or dominant rat [126,127]. This effect probably does not occur immediately and requires some repeated calling, and the opposite might not be true, i.e., the lack of emission of appeasement calls will not necessarily increase attacks of the aggressor, as it was observed in studies with devocalized rats in which deprivation of ultrasonic signals failed to increase aggressive behavior of the attackers [136]. In this experiment, however, rats could not communicate by any type of calls. Appeasement 22 kHz calls may also be emitted to prevent attack in establishing a dominant–submissive relationship. It was observed that a face-to-face encounter with a dominant rat immediately induced the emission of 22 kHz vocalizations in the submissive rat (usually smaller in size) [137].

It was also suggested that during play behavior (both in juvenile and adult rats), the emission of 50 kHz calls may not only be a play signal but also an appeasement signal that de-escalates agonistic behavior during play and prevents aggressive outcomes, which can happen particularly in playing rats that are unfamiliar to each other [99,138].

Finally, it should be mentioned that audible squealing (sonic threat calls) in young rats that are emitted after bites as pain signals, have been also suggested in the past to have an appeasement effect [58] (p. 126).

### 2.5. Functions of Vocalization in Reproductive Behavior

#### 2.5.1. Mating Function

The emission of ultrasonic calls was well studied in the mating and reproductive behavior of rodents. This is a complex and partially ritualized behavior, so the emission of many types of calls were observed. These vocalizations play a mating function, i.e., they contribute to the regulation of the selection of partners, soliciting sexual contact, and initiating copulation. Fifty kilohertz vocalizations are emitted mostly during solicitation and mounting activity, while 22 kHz are emitted by males during the postejaculatory refractory period [73,123]. Male rats emit 50 kHz before successful mating, which may facilitate female responsiveness because females were less responsive when paired with a devocalized male [123,139]. Females also emit ultrasonic calls before copulation that were suggested to play a regulatory role in mating [140,141]. The emission of 50 kHz calls was dependent on female sex hormones because ovariectomized females exhibited few, if any, of the vocalizations [142] and their responses were graded depending on the hormonal condition [143].

Some recent studies, however, could not fully confirm the behavioral role of 50 kHz vocalization during mating [144,145]. Many factors could cause a lack of this response, such as rat strain, too-frequent repetition of tests, rat experience, or stress. Ultrasonic communication during mating is highly dependent on the gonadal status of both partners. For instance, it was found that females produced more 50 kHz ultrasonic vocalizations to intact males than to castrated males but produced similar numbers of calls to both relevant groups of females [146]. Using a playback paradigm, the role of ultrasonic calls in mating was studied in another laboratory, and it was concluded that female rats displayed high levels of social approach behavior in response to the playback of male, 50 kHz ultrasonic vocalizations and did not respond to amplitude-matched white noise [147]. The emission of 50 kHz calls plays an important role in establishing social proximity [147], and it may be concluded that is important in the regulation of mating behavior.

#### 2.5.2. Social Detachment Function

Male postejaculatory 22 kHz vocalizations are particularly long calls [148]. However, unlike other types of 22 kHz vocalizations, they may have some limited frequency modulation, particularly in the medial and terminal fragments of the call. These calls were postulated to represent a different emotional state of the rat than during the emission of flat-type alarm 22 kHz vocalizations in other situations [149]. The emission of shorter 22 kHz calls than the postejaculatory calls may appear during mating and these calls are associated with unsuccessful intromissions or failed mountings [148], so aversive situations to males.

The postejaculatory 22 kHz vocalizations represent a state of behavioral inhibition with prolonged immobility, a withdrawn or socially depressed state, and an absolute refractory period with “desist-contact” function ([149,150,151,152]. During the postejaculatory calling state, rat males do not copulate [153]. It was suggested that the postejaculatory calls have function of keeping the females away [123,150]. It has been observed that experienced females leave the male during the emission of these calls [154]. Contrary to that, the prolonged presence of females together with males increased the duration of the emission of postejaculatory 22 kHz calls [152,155]. This notion that females would avoid male during emission of postejaculatory 22 kHz calls was, however, not always detected [156]. Many factors may influence this behavior and sexual experience is one of them. Nevertheless, the 22 kHz ultrasonic vocalizations in this situation may have a social detachment function or social disaffiliation function, i.e., a role opposite to the affiliative function. This function would be equivalent to the appeasement function in agonistic encounters, discussed above.

Acoustic analysis of the postejaculatory of 22 kHz vocalizations emitted by males during sexual contacts, or intended contacts, revealed that the calls are heterogenous and consist of long flat 22 kHz calls (20–35 kHz range) during the postejaculatory period, and anther class of 22 kHz calls. The other class of calls has higher sound frequency (23–45 kHz range) and some modulated frequency components that were observed during encounters of males with a female in a cage with a physical, perforated barrier, where animals could not have physical contact [157]. This last precopulatory category of calls would be compatible with a negative state of frustration caused by the presence of an inaccessible female, and not as a social detachment function [157] (for more details, see Section 2.8 and Section 3.5).

### 2.6. Functions of Vocalization in Ingestive Behavior

#### 2.6.1. Alimentary Function

The phenomenon of the social transmission of information about food has been studied in rats for some time [158,159,160]. The results indicated that the transfer occurs via olfactory cues, and the observers rely on smelling the breath of the demonstrator rat, who has had direct contact with the food [161,162]. Since rats are very vocal in social interactions, the question as to whether rats can convey food preferences via ultrasonic vocalizations was still open and was studied first in female rats. The results suggested that ultrasonic vocalizations do not play role in this communication because information conveyed by the demonstrator rat had no significant influence on the food choices of the observers when the rats were devocalized [163].

This experiment, however, could not fully explain the mechanism of the vocal transmission of feeding information in rats, and the possibility of the vocal transmission of food preferences was recently raised again [164]. Some clues may come from studies performed on female mice, showing that the observer mouse emits ultrasonic vocalizations toward the demonstrator mouse that has been recently fed, but these vocalizations are dependent on the motivational state of the observer. Non-deprived animals emitted more calls toward demonstrators that were fed on palatable food, while food-deprived animals vocalized more to mice that were fed on any food regardless of its palatability [165]. These calls facilitated the proximity of the mice; however, the exact motivation for the emission of these calls and their communicative value need further studies.

These and other experiments justify distinguishing the category of alimentary calls in rats. In an earlier study, the structure of rat vocalizations emitted by pairs of rats (and recorded in pairs) was studied and categorized, and then the categories were assigned to specific behaviors [166]. Three clusters of ultrasonic calls were identified, roughly referring to frequency-modulated 50 kHz calls, flat 50 kHz calls, and 22 kHz calls. It is of interest that the middle cluster, which was equivalent to the flat type, 50 kHz vocalization (with frequency range between 35 and 55 kHz), contained calls that were emitted mostly during feeding and their emission was consistent [166]. The communicative role of flat versus frequency-modulated 50 kHz vocalizations is dissimilar. For example, during experiments with the self-administered playback of 50 kHz vocalizations, rats reliably self-administered frequency-modulated 50 kHz calls with trills but not the flat 50 kHz calls [73].

#### 2.6.2. Food Provisioning Function

In another recent study, pairs of rats were tested in a mutual food-provisioning task [167]. Firstly, it was found that receiver rats emitted 50 kHz ultrasonic vocalizations toward their donor partners, and the donors provided food to the receivers by pulling a tray with a treat toward the partner rat without a reward for themselves. This food delivery was done in a proportional way to the receivers’ communication [167]. These results justify distinguishing a category of ultrasonic calls that have a food-provisioning function. Further research, however, is needed to better understand the type and behavioral role of these calls. It is not clear whether rats were only expressing the need and/or requesting food, or advertising sources of food to other rats.

It seems, however, that the 50 kHz vocalizations in this situation have positive signaling value, i.e., they would be associated with appetitive expectation of food, approach, and eating behavior. On the other hand, it has been shown in the past that alarming 22 kHz vocalizations had an opposite effect on eating behavior. When 22 kHz alarm calls signaled the proximity of a predator (cat), eating behavior was inhibited for up to 2 h [168]. When the alarm subsided, rats emerged from their burrows and resumed eating, but eating bouts were shortened and frequently interrupted by careful observing of the environment [169]. Thus, alarm calls discourage eating, so they have the opposite function to the food provisioning one.

### 2.7. Functions of Vocalization in Defense against External Threat

#### 2.7.1. Predator Alarming Function

The most known and well-studied function of rat ultrasonic vocalizations is the alarming function. It evolved as one of the fundamental antipredator behaviors [168,169,170,171]. The alarm calls are long-duration 22 kHz vocalizations with relatively constant, i.e., unmodulated, sound frequency and are emitted for a prolonged time, call after call, after detection of the predator and for about 30 min after the predator has left [170,171]. The alarm calls are directed to the members of the entire social group (audience effect) and related to the approaching danger. However, the social effect may not be present, i.e., a rat may emit alarm calls when it is isolated from the group (particularly in the laboratory) or when it may not know where other conspecifics are [172]. Alarm calls are emitted from the place of a relative safety (not in the immediate reach of the predator when the fear response appears) and are not directed to the predator [173]. The alarm vocalizations usually cause a freezing response of the recipient rats, or their escape to the burrows. This effect was reproduced in an experimental situation in which rats that were chased by a fast-moving object (as a potential predator) showed an escape response with the emission of 22 kHz vocalizations and freezing episodes [174].

It has been documented that rats are also highly afraid of predator odors and consistently respond to them with defensive behavior [175]. The odors originate from predators’ skin and fur, urine, feces, and anal gland secretions [176]. Rats respond to odors of many predators (e.g., cat, fox, or lion) but the alarm response to the cat’s odor is the strongest [177,178]. When rats were placed in a protective tube within the cage of a predator, they reliably emitted 22 kHz alarm calls to the odor of a cat but emitted only a few calls to that of a snake, and no calls to the odor of a ferret or a control, clean cage [179]. Interestingly, rats did not raise an alarm to the odor of ferrets, which are large carnivores and pose a danger to rats. Ferrets, related to polecats and weasels, however, have been domesticated for a very long time, probably for 2000 years [180]. They live with humans and this could cause some changes in their bodily odors, and it may explain why rats did not recognized the ferret’s odor as a threat in this study.

Some analyses of alarming vocalizations of many species led initially to the suggestion that alarm calls evolved to be communicated to predators [181], but other observations have not supported this view. Although cats and other large land predators can hear ultrasonic calls of rats, it was postulated that the ultrasonic alarm calls in rats evolved to protect them from birds of prey (a couple of hundred of species of them) that are the most dangerous predators to rodents [182]. This protection against predators could evolve by adaptation or by exaptation, i.e., by use of naturally preexisting ultrasonic sounds produced by narrow airways for disguised communication. Birds of prey cannot hear ultrasonic calls and their usual audibility is between 1 and 4 kHz (with the exception of the tawny owl, hearing up to 20 kHz) [183]. Thus, communication in the ultrasonic range is adaptive and protects rats. When cornered by a cat, rats have the capacity to defend themselves and often do that successfully as it was indirectly confirmed by a recent publication providing evidence that feral cats were ineffective in hunting for urban rats [184]. Rats have very sharp incisors, and their bites leave deep and not-well-healing wounds infected by bacteria carried by rats [185]. However, rats are defenseless against fast moving birds of prey, which can reach a velocity of 52–70 m/s in extreme situations, as measured for the falcon [186], or developed adaptation for a silent flight as that one of owls [187].

Behavioral analysis of the emission of 22 kHz alarm calls and audible squeals that were emitted in dangerous confrontations with predators or large mammals led to the conclusion that ultrasonic 22 kHz calls are directed to other rats and are associated with an audience effect, while audible squeals are emitted as warning calls directly to predators and other large animals (including humans) and do not require the presence of other conspecifics [173].

The alarming function of 22 kHz ultrasonic calls does not serve individual protection but is a form of social anti-predator defense, i.e., emission of these calls warns the entire social group. This social behavior was regarded as a higher order of defense [18,188]. Hearing the alarm, rats will respond (usually by escape and hiding) regardless of whether the individual colony members have detected or not detected the presence of the predator [171]. Once initiated (usually by alpha male), the alarm is maintained and emitted repeatedly by everyone in the colony for a prolonged time as studied in the visible burrow system [171]. At that time, animals reduce their activity or freeze as it was studied in many independent experiments with the replay of 22 kHz calls [178,189,190].

#### 2.7.2. Alarming-Warning Function

Alarming 22 kHz calls are emitted not only in response to predators, but also when rats encounter any other direct danger. Thus, the alarm calls serve as a general danger signal, so they possess an alarming–warning function to other rats. The alarm calls may be emitted in contact with an unfamiliar human [191,192], in response to a sudden noise (startling acoustic stimulus) [193,194], an unpredictable tactile stimulus with a hissing sound (air-puff) [188,195], or an electric foot shock or tail shock [196,197,198,199,200,201]. Thus, the semiotic value of the 22 kHz calls in these situations is not related to the predator but is sent as a signal of a general but real and present danger, even though the animal might not fully recognize the nature of the danger (e.g., air-puff) and the danger may not necessarily relate to other rats. These alarming–warning calls are like predator alarm calls and are often interpreted as alarm calls but they differ from them by the circumstances. The alarming–warning category of calls was usually studied in single rats in laboratory cage conditions.

#### 2.7.3. Security Function

It has been also suggested that the emission of 22 kHz vocalizations may serve as a signal of potential danger that is not actually perceived at a given time by the rat. This emission would be initiated by a special motivational system, termed security motivation system, which was well studied in rats [202,203,204]. It was argued that this system evolved to cope with unpredictable environmental risks, uncertainty, and potential, but not directly observed or detected, dangers [205]. In this situation, the emission of 22 kHz calls would have a security function. Such alarm 22 kHz vocalizations would serve as precautionary signaling of a potential danger, i.e., as an apprehension signal before any danger appeared [205]. The triggering events could be cues originating from the similarity of an environmental situation to the past aversive events, or the lack of stimuli that the animal would expect to detect (e.g., disappearance of a nearby predator), or some weak or new stimuli unknown to the animal.

The emission of calls from security motivation has not been described; however, there are some fragmentary observations suggesting such motivation. We observed such an emission in the laboratory, when a rat sitting quietly and silently in a cage suddenly started emitting 22 kHz calls without any provocation or other stimuli detectable to humans. The category of anticipatory calls has already been demonstrated for vocalizations expressing a positive emotional state associated with drugs of abuse [206]. Security motivation signaling requires further systematic studies.

All the functions described in the subsections above are associated with significant endocrine and autonomic changes, such as release of ACTH, changes in blood pressure, heart rate, body temperature, and respiration rate and confirm the stressful and emotional nature of situations associated with the emissions of ultrasonic vocalizations [207,208,209,210]. Although initial recordings of heart rate during the playback of ultrasonic calls did not detect changes in heart rate [211], more detailed and frequent sampling of heart rate detected changes caused by the playback. It is interesting that receivers of the ultrasonic calls develop relevant emotional arousal with autonomic changes [212]. Playback of 22 kHz vocalizations decreased heart rate in the receiver rats, while the playback of 50 kHz calls increased the rats’ heart rate. These effects were stronger in singly housed rats as compared to pair-housed rats [212].

### 2.8. Functions of Vocalization in Expressing Internal Discomfort and Frustration

#### Frustration Expression Function

It should be also mentioned that the emission of 22 kHz vocalizations may express an anhedonic state, originating from other situations than external predatory or other dangers. The best example is a cycle of positive (euphoria) and negative (dysphoria) affective events observed in organisms addicted to drugs of abuse. It has been observed that during the withdrawal phase from a drug (e.g., from cocaine, heroin, amphetamine, opiates, and ethanol), rats will emit large numbers of 22 kHz vocalizations for many hours after discontinuation of the drug [213,214,215,216,217]. The emission of 22 kHz vocalizations signaled a negative affective state (dysphoria, anhedonia, or frustration), and the calls appeared right at the time when drug levels in the rat body started decreasing, even between binges of self-administration and later during the withdrawal state [217]. This negative emotional state was signaled by 22 kHz calls and initiated by deprivation of the expected drug delivery. This shift from a positive to negative emotional state was suggested to present a salient motivational factor for seeking more drugs, which is well known from the behavior of human drug addicts [218]. As it is known from studies on human patients, the withdrawal state is a powerful psychopathological state and even in former addicts with extinguished drug-seeking behavior, the state can be reversed and cause strong craving relapse when subjects are exposed to environmental situations previously paired with drug-taking situations [219].

In rats, when the addictive drug is not available, the emission of 22 kHz calls signals dysphoric frustration, so the calls may have a frustration expression function. A similar situation with the emission of 22 kHz vocalizations as expression of frustration and frustration-induced anxiety was also reported in rats. This type of call was observed during sexual contacts between male and female rats when the physical contact between the animals was prevented by three physical barriers with not-aligned holes that allowed for olfactory, visual, and auditory contact but not physical, tactile contact. Male rats emitted long 22 kHz vocalizations but with altered frequency structures (for acoustic details, see Section 2.5.2, above) as compared to postejaculatory 22 kHz calls. These calls were associated with the exploration of holes in the barriers and were compatible with irritation and frustration [157]. These frustration calls were also observed in rat sexual encounters during unsuccessful mounting attempts or failed intromissions [148].

## 3. Vocalization as Expression of Emotional Arousal

### 3.1. All ultrasonic Vocalizations Are Emotional Expressions

#### 3.1.1. Characteristics of Vocal Expression of Emotional Arousal

The major functions played by rat ultrasonic vocalizations are summarized in Table 1. The circumstances causing animals to emit vocalizations lead to several conclusions. The emission of ultrasonic calls for all the functions has an emotional nature, so the calls represent the expression of emotional arousal with motivation to influence the situation, which instigated this arousal. Although the particular behavioral circumstances differ, these states have common features typical for emotional response, such as increased arousal, prolonged and focused attention, increased muscular tension and/or motor activity, emission of vocalizations, increased activity of the autonomic and endocrine systems, and certain persistence of the response [220,221]. It could be argued that vocal expression of emotion for its own sake does not exist. Animals express their emotional states vocally in specific situations only as means of changing or modifying the social and biological circumstances that induced these states. For this reason, the function of expressing emotion as an independent category was not distinguished in this review.

The understanding that rodent vocalization is produced by arousal was first clearly stated in 1974 [222]. The notion that the emission of 22 kHz ultrasonic calls in rats specifically expresses emotional arousal is also old and was postulated over 30 years ago [223,224]. In later studies, the vocal expression of emotional states in rats was confirmed for infant calls and for adults emitting 22 kHz or 50 kHz calls by many laboratories [23,30,43,214,221,225,226,227,228,229,230].

In adult rats, ultrasonic vocalizations express two different basic emotional states: an aversive state (displeasure) or appetitive state (pleasure). Each of the functions listed in Table 1 may be assigned to one or the other state (or both) and is labeled in the table as a positive or negative state. Vocal signaling in infants is interpreted as distress and the expression of an early anxiety state [23]. Early infant vocal signaling is a reflexive and automatic process because pups do not hear calls until Postnatal Day 12 [231].

Vocalizations evolved as a social adaptive strategy and are directed to other members of the social group [205]. Size of the social group, its organization and complexity will have influence on the vocal repertoire (for review, see [205]). The production of ultrasonic vocalizations is a complex process from the brain control point of view. Complicated sound production by the larynx in rodents (as in all mammals) is simultaneously coupled with the control of respiration and heart rate [232,233,234]. Calling is energetically costly, particularly for prolonged vocalizations, as it was directly documented in frogs continuously vocalizing for 2–3 h [235]. Thus, prolonged vocalizations are emitted only as a necessary activity initiated by growing emotional arousal.

#### 3.1.2. Initiation of Emotional Arousal by the Brain

Emotional arousal is a powerful and extensive central process that changes the state of the entire brain [236] and as a result, emotional arousal leads to functional changes in the entire body, from autonomic adjustments to changes in the motor and sensory systems [237,238] and changes in animal behavior. Some manifestations of emotional arousal and the emerging emotional state might be marginal [239]; others, such as the emission of vocalizations, are powerful and carry significant semiotic value to conspecifics. The semiotic content of calls does not serve as sending specific (lexical) information, but it is always an emotional instrument of influence on other conspecifics to control their behaviors [222,240]; also, it is a behavioral plea for change, even if the change is impossible. From these reasons, it is not possible to directly translate the semiotic content of rats’ emotive vocalizations to human lexical language, an idea that was formulated for the first time by McLean [241].

Emotional arousal is triggered by innate brainstem limbic mechanisms in response to incoming environmental stimuli and cues (complex stimuli) or lack of thereof, although the exact mechanism of this initiation is not fully known. These phylogenetically old mechanisms are located in the medial brainstem reticular core [242,243], and more precisely, in the oldest part of the idiodendritic core with neurons having overlapping dendritic fields. The reticular core of the brainstem reaches up to the diencephalon, the hypothalamus, and the septum [21]. This extensive system remained relatively unchanged in the process of evolution and deals with arriving afferent signals of heterogenous origin [244]. The core is part of the larger structure, reticular formation, stretching from the spinal cord to septum, although it is lacking precise neuroanatomical delineation [21]. The reticular core evolved for broadly understood sensorimotor integration and control of behavior [245].

The most general function of the reticular core was described as an activity leading to adaptive stability of the organism [21]. The generation of emotional arousal in relevant situations serves this function. Nauta understood the adaptive stability as an analog of homeostatic mechanisms. While classical homeostasis is concerned with stability of the internal environment of the organism, adaptive stability pertains to the stability of the relationships between the organism and the external environment [21]. The emission of vocalization is one of the fundamental tools in interacting with this environment (mostly social environment). In recent decades of studies on emotional states, the attention has been diverted from the brainstem to numerous other structures, including the neocortex; however, recently, the critical importance of the brainstem in the initial generation of emotional arousal and emotional state has been again acknowledged [246].

### 3.2. Dichotomy of Emotional Arousal

#### 3.2.1. Limitations of Infantile Vocalizations as Relics of Paleomammalian Communication

Very young rat pups express only primeval aversive states associated with a basic self-preservation function. This aversive arousal is based on early parasympathetic regulation and most probably evolved before the evolution of the sympathetic control [8,247]. The pups’ brain is immature, and growth of the myelinated innervation of the larynx is not yet completed [248]. The developing myelinated recurrent laryngeal nerves reach the larynx by Postnatal Day 15, and the formation of neuromuscular junctions in the larynx is not finished sooner than Postnatal Day 19 [249]. Only after this innervation emerges can intrinsic laryngeal muscles fully develop [250]. This happens just about the time when pups stop emitting juvenile isolation calls and begin the transition to adult forms of vocalization (about the Postnatal Days 21–23) (unpublished observations and [251]). This stage also coincides with the development of homoiothermy [252].

Thus, without full laryngeal innervation, rat infants are not capable of emitting adult-type vocalizations and initially rely on inborn mechanisms of poorly regulated, heterogenous broadband calling. Since the myelinated ventral vagal complex that innervates the larynx evolved as the last component of the autonomic nervous system and is responsible for the generation of adult ultrasonic calls [8], one may speculate that infantile isolation calls may be similar to primitive vocal communication at the paleomammalian stage, i.e., mammalian ancestors’ stage of evolution. The term paleomammalian brain was coined by McLean [241] and this evolutionary ancestral brain was identified with the basic limbic system. At this earliest stage, only negative arousal was signaled; hence, the infantile calls have only negative valence.

#### 3.2.2. Dichotomy of Adult Emotional Arousal Systems and Emotional Signaling

The adult rat ultrasonic vocalizations fall into two categories of different emotional valences and are labeled as 22 kHz and 50 kHz calls. These two categories of calls (with some limited variation of frequencies within each category) differ by 2–10-fold in all acoustic parameters [236] so they are easily discriminated by rats. Although many acoustic features of rat ultrasonic vocalizations may play a role in this discrimination, the sound frequency band proved to be the most informative and critical for this discrimination [253]. The mean sound frequency of any vocalization and any valence was approximately three times more likely to serve for the proper discrimination of calls than frequency modulation within the call, and 6.5 times more likely to discriminate a call than that based on its duration [253]. This call discrimination is biologically important because the 22 kHz and 50 kHz vocalizations signal two different emotional states that should be recognized by rats.

**Figure 1:**
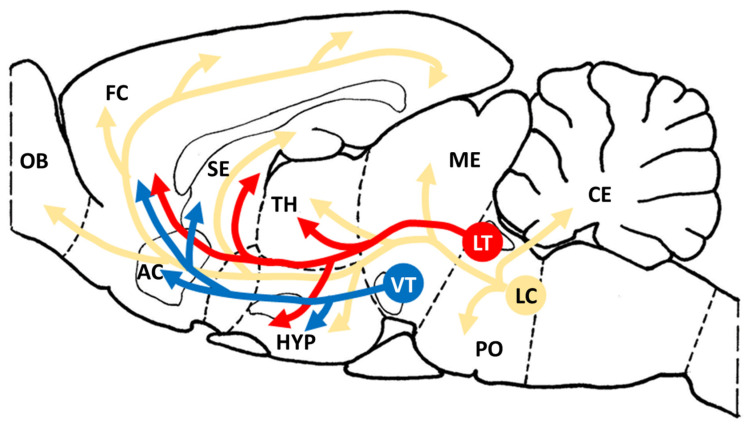
The diagram presents a rough outline and relationship between the ascending cognitive arousal system and two emotional arousal systems in the rat brain. The cognitive arousal system (yellow arrows) originates from the locus coeruleus (LC), releases norepinephrine and targets most of the brain but particularly the neocortex. The mesolimbic aversive emotional arousal system (red arrows) originates from the laterodorsal tegmental nucleus (LT) and targets extensive limbic regions through hypothalamus (HYP) to lateral septum (SE) and releases acetylcholine. The mesolimbic appetitive emotional arousal system (blue arrows) originates from the ventral tegmental area (VT) and targets predominantly the nucleus accumbens (AC) and neighboring regions and releases dopamine. Both mesolimbic arousal systems are most probably also reaching the frontal cortex (FC). The diagram shows only the essential parts of these two emotional arousal systems, which represent relevant functional fragments of all cholinergic and dopaminergic neurons in the brain. It is clear at the first glance that the ascending emotional arousal systems are targeting predominantly subcortical limbic regions. Other abbreviations: CE—cerebellum, ME—mesencephalon, OB—olfactory bulb, PO—pons, TH—thalamus.

The aversive 22 kHz vocalizations are initiated by the ascending mesolimbic cholinergic system, while the appetitive 50 kHz vocalizations are initiated by the ascending mesolimbic dopaminergic system [254]. Unlike the cognitive arousal system, these two emotional arousal systems are targeting predominantly subcortical, limbic regions (Figure 1). This dichotomy in mesolimbic innervation evolved as an extension of the dichotomy in the autonomic nervous system that forms sympathetic and parasympathetic divisions. Two parallel, ascending mesolimbic emotional arousal systems have different and antagonistic functions, which prepare the animal for two different and behaviorally opposite outcomes, i.e., for danger in an aversive situation (negative state), and for affiliation and hedonia in an appetitive situation (positive state). Thus, the valence of the emotional arousal is mostly predetermined by the dominating activity of the type of the ascending mesolimbic system that initiates it.

#### 3.2.3. Aversive and Appetitive Arousals Are Antagonistic Processes

The existence of two emotional arousal systems leads to the conclusion that aversive and appetitive arousals are mutually exclusive. The aversive and appetitive behaviors are controlled by different mechanisms, are based on different neurotransmitters, and form separate processes that cannot guide animal behavior at the same time. This dichotomy seems to be a general rule not only in vertebrates but also in invertebrates as it was recently shown for crabs [255]; otherwise, it would be maladaptive.

In rats, pharmacological experiments in which the arousal states were induced by intracerebral injections of cholinergic or dopaminergic agents provided evidence that the aversive state and appetitive state are antagonistic processes. The results showed that pharmacological initiation of an aversive state signaled by the emission of 22 kHz calls was significantly attenuated by a subsequent direct pharmacological initiation of the opposite, appetitive state in the brains of the same animals [256,257]. Not only the aversive cholinergic state may be inhibited by the activity of the dopaminergic system but there is evidence for the oppositive inhibition. In anesthetized rats, the identified dopamine neurons in the ventral tegmental area were all inhibited by an aversive stimulus [258]. In behavioral tests with the measurement of rat locomotor activity, a similar result was obtained. An amphetamine-induced increase in locomotor activity was antagonized by intracerebral application of carbachol into the anterior preoptic–hypothalamic region (part of the terminal fields of the ascending mesolimbic cholinergic system) [259]. This antagonism between opposite emotional states provides researchers with an additional way of assessing changes in the emotional valence. Thus, a rapid decrease in the emission of 50 kHz calls or a rapid decrease in the emission of 22 kHz calls may be interpreted as an aversive or appetitive shift, respectively.

Although the initiation of a positive emotional state (dopamine) functionally antagonizes the initiation of a negative emotional state (acetylcholine) in rats, these two systems do not work in a mirror-image way. Pharmacological antagonism of the mesolimbic dopaminergic system did not automatically increase the emission of 22 kHz vocalizations. On the other hand, cholinergic overstimulation of the aversive system with abundant emission of 22 kHz vocalizations caused a delayed rebound effect in the form of the spontaneous generation of 50 kHz calls in a proportional way to the intensity of the initial aversive response [260]. Moreover, the rebound emission of 50 kHz vocalizations was entirely blocked by haloperidol, proving that the emission of 50 kHz from whatever reason is generated by dopamine [260]. The question arises as to how cholinergic stimulation can initiate a delayed rebound with an underlying dopaminergic mechanism.

The rebound could be explained by the activity of a branch of the ascending cholinergic system from the laterodorsal tegmental nucleus to the ventral tegmental area [261]. These cholinergic fibers terminate on dopaminergic neurons of the mesolimbic (mesoaccumbens) dopaminergic system and have excitatory effects [262]. Cholinergic activation of the ventral tegmental dopamine neurons was shown to occur by cholinergic M5 type of muscarinic cholinergic receptors and caused the release of dopamine in the nucleus accumbens, particularly in a delayed phase of the prolonged release of dopamine [263]. This mechanism could explain the appearance of the 50 kHz rebound phenomenon. The exact role of the cholinergic input to the ventral tegmental area is not yet clear, but this is a different sub-system than that one for the initiation of the negative emotional arousal. In the aversive arousal, D1, D2, and D3 dopaminergic receptors are involved [62,264] while the cholinergic input to the tegmental dopaminergic neurons utilizes D5 dopamine receptors with a different pharmacological characteristic. Prolonged activity of the cholinergic neurons of the laterodorsal tegmental nucleus, as that one induced by long-lasting action of cholinergic agents, seems to initiate “a break” by activating the dopaminergic system, which gradually takes over.

On the other hand, the opposite situation may happen with the dopaminergic system. Prolonged stimulation of the dopamine neurons in the ventral tegmental area may decrease their activity and result in aversive arousal. Recent results have shown that the loop between the nucleus accumbens and the ventral tegmental area may be involved in inhibiting the activity of ventral tegmental dopaminergic neurons depending on the duration of stimulation. In the most recent study, brief optogenetic stimulation of the accumbens medium spiny neurons increased ventral tegmental neuronal activity and increased rewarding responses while prolonged stimulation of these neurons induced aversion and decreased rewarding effects [265]. A functional relationship between these two mesolimbic systems and the mechanism of the initiation of emotional arousal are complex and need further studies.

#### 3.2.4. Emotional Arousal versus Cognitive Arousal

Emotional arousal is a separate process from cognitive arousal that is carried out by the classical reticular activating system innervating entire neocortex by noradrenergic axons [266] (see Figure 1, yellow arrows). These two arousal modes (emotional and cognitive) are functionally coupled together and can directly interact with each other, at least in the brainstem [267]. Emotional and cognitive arousal work in concert but target different structures (limbic structures and only limited frontal neocortical regions versus vast areas of neocortex).

Since the predominantly noradrenergic cognitive arousal maintains the awake state and vigilance [268], it is expected that this system needs to be active to allow emotional arousal to perform its function. This was demonstrated in a pharmacological experiment. During amphetamine-induced emotional arousal with the emission of vocalizations, pharmacologic antagonism of selected subtypes of receptors of the noradrenergic system significantly decreased the emission of 50 kHz calls or selectively decreased some subtypes of 50 kHz calls, such as trill calls, the most characteristic components of emotional expression [269]. In another study with the emission of 50 kHz calls by male rats in response to a female (initially present but removed for recordings), noradrenergic agonists led to an increase in the intensity and duration of ultrasonic calls while antagonists reduced the call rate, intensity, and bandwidth of 50 kHz calls [270].

There is not much research on this topic that is published but it seems that the role of emotional arousal (positive or negative) is to enhance neocortical information processing for emotionally important stimuli (salient stimuli) and, at the same time, decrease the processing of stimuli that are not biologically important at that time [271]. This process most likely occurs right in the brainstem by the interaction of the ascending arousal systems. In electrophysiological studies, it was observed some time ago that the ascending noradrenergic system exerts a tonic influence on the neocortex to maintain the waking state; however, the ascending cholinergic system provides additional input in a phasic manner in response to novel, unfamiliar, or threatening stimuli (the emotional component) [272,273].

### 3.3. Pharmacology of the Systems for the Initiation of Emotional Arousal

There are several diffuse ascending systems that originate from the brainstem that are involved in the generation and/or modulation of arousal, and the concomitant general animal state and functioning of the whole brain. All these systems have extensive ascending axon pathways reaching most of the brain, although the density of innervation varies among structures. Each of these systems utilizes a single main neurotransmitter that is massively released during activity mostly by numerous varicosities, suggesting a volume transmission in vast areas of the brain [274]. The following major systems have been identified as arising from the brainstem and associated with changes in brain functions and arousal: (1) the noradrenergic system arising from the locus coeruleus [275]; (2) the ventral dopaminergic system arising from the ventral tegmental area and substantia nigra [276,277]; (3) the brainstem cholinergic system arising predominantly from the laterodorsal tegmental nucleus and pedunculopontine nucleus of pontomesencephalic reticular formation [278]; (4) the serotonergic system arising from raphe nuclei [279]; (5) the histaminergic system arising from basal hypothalamus, mostly tuberomammillary nucleus of the posterior hypothalamus [280]; and (6) the orexinergic system arising from neurons in the lateral and posterior hypothalamus [281,282]. The volume of literature published on these ascending systems is particularly large, so detailed discussion of these systems and their projections is beyond the scope of this review. Although all these ascending systems are, directly or indirectly, involved in emotional mechanisms, there are only two basic systems that are critical in the initiation of emotional arousal with the emission of vocalization.

As demonstrated in the previous sections, emotional arousal in the rat’s overt behavior is signaled by the emission of ultrasonic vocalizations. The following question arose: which of the six ascending systems mentioned above, when stimulated, can quickly and efficiently induce species-specific vocalizations and other behavioral manifestations of emotional arousal? It appeared, in rats, that the direct cholinergic stimulation of vast areas of the medial diencephalic and forebrain structures, up to the lateral septum, induced abundant aversive 22 kHz vocalization with other signs of a negative emotional state (such as decrease in activity, freezing, crouching, signs of anxiety, etc.) [283,284,285,286,287]. The emission of 22 kHz calls was dose-dependent and antagonized by atropine, suggesting muscarinic mechanism. This aversive system is marked with red arrows in Figure 1. On the other hand, direct dopaminergic stimulation of the nucleus accumbens and adjoining regions uniformly induced abundant emission of 50 kHz vocalizations with increased locomotor activity [105,288,289,290,291]. The response was dose-dependent and antagonized by raclopride, suggesting, at least some, dopamine D2 receptor involvement. This appetitive system is marked with blue arrows in Figure 1.

The emission of 50 kHz calls could also be induced from the hypothalamic–preoptic regions by intracerebral glutamate, but this emission was dependent on dopaminergic neurotransmission and was antagonized by haloperidol [289]. Additionally, emission of 22 kHz calls could be released by direct glutamate stimulation of the laterodorsal tegmental nucleus, and this emission was antagonized by atropine [285]. Although glutamate can initiate 22 kHz or 50 kHz vocalizations, their generation and emission remain dependent on the dopaminergic system for 50 kHz calls or on the cholinergic system for 22 kHz calls.

For comparison, numerous pharmacological–behavioral studies were unable to unconditionally induce emotional states with continuous emission of ultrasonic calls after direct intracerebral application of neurotransmitters utilized by any of the other ascending brainstem systems. Intracerebral application of norepinephrine [269,270], nicotine [292,293], serotonin [294,295], or application of orexin [296,297] appeared ineffective in inducing emotional arousal with the emission of ultrasonic calls. All the mentioned neuroactive agents, however, had a modulatory effect on the ongoing emissions of ultrasonic calls that were induced naturally or pharmacologically. It was, therefore, concluded that only the ascending mesolimbic cholinergic and dopaminergic systems have the capacity of initiating emotional arousal that leads to overt behavioral manifestations with the repeated emission of ultrasonic vocalizations.

It may be further concluded that the magnitude of the emotional arousal is proportional to the amount of released neurotransmitter—acetylcholine for the aversive state, or dopamine for the appetitive state—because emissions of pharmacologically-induced vocalizations were proportional to the doses of injected agents that initiated the arousal [105,283]. Transmitters of all other extensive ascending systems have only a modulatory influence on the two basic systems (dopaminergic and cholinergic).

Considering the anatomy of mammalian brains, it may be postulated that these two parallel and behaviorally opposite emotional arousal systems are homologous systems in the brains of all mammalian species and are universally responsible for two basic emotional states: positive (appetitive) or negative (aversive). As for species other than rats, so far, only the aversive arousal state with a consistent, growling vocalization was thoroughly studied in cats, and the results were similar to those for the rat species, with a homolog ascending cholinergic mesolimbic system, comparable terminal fields, comparable pharmacology of aversive vocalization, and comparable emotional valence (for a full review of studies on cats and comparison of the results with those on rats, see [284]).

### 3.4. Transition of Infant Isolation Calls to Adult Calls

#### 3.4.1. Development of Rat Auditory Cortex

In addition to self-preservation and protective functions, infant calling also serves to develop the mother–infant bond [298]. The initial development of pup vocalizations is a highly autonomous process that is not much influenced by external stimuli [299,300]. The question arises of how the infant responds to the mother’s calls and how its vocalizations could rapidly change from automatic infantile calls to “meaningful” and behaviorally relevant signals within several days of development. A partial answer to this question may be provided by the mechanisms of brain development itself, and particularly, capabilities of the pups’ auditory cortex.

The cortical auditory representation of ultrasounds contained in ultrasonic vocalizations is particularly well developed in the primary auditory area (A1) of the rat cortex [301]. Close to 40% of the primary auditory cortical (A1) responses represents an octave-wide band for critical sound frequencies used in ultrasonic vocalization (32–64 kHz) (i.e., all 50 kHz calls and some 22 kHz calls), while the responses to other sound bands that are below 32 kHz form only 20% of the A1. The group of frequencies for 22 kHz calls is somewhere at the border of these two cortical regions. The 32–64 kHz frequency bin occupies more surface area of the auditory cortex than any other single bin, from 1 to 32 kHz [301]. The adult rat auditory cortex has a clear overrepresentation of neurons responding to sounds characteristic for ultrasonic calls.

The overrepresentation of ultrasounds in the rat auditory cortex, however, needs early life acoustic experience and rapidly develops from the third week of postnatal life [301]. Each day of postnatal life makes a big difference in the development of cortical representation and, for example, the difference between postnatal Day 20 and 21 makes a highly significant increase in the cortical representation for sounds of about 60 kHz [301]. This is a developmental process, but it is based on exposure to ultrasounds and their perception. Hearing loss caused by ear ligation significantly prevented the developmental increase in the percentage of the A1 auditory region for sounds of 32–64 kHz [301]. This experiment may illustrate how early in a rat infant’s life the acoustic system develops and most probably makes already early associations between vocalizations and some behavioral situations.

Another question was raised of how the rat brain can distinguish among biologically important sounds (calls) and other unimportant environmental sounds. Numerous studies have shown that sounds that are overrepresented in the acoustic cortex of rats are those that are in the ethological range and are frequently repeated within the critical period of the development. This repetition-dependent cortical plasticity generates the overrepresentation, i.e., more cortical neurons are tuned to these sounds [302].

#### 3.4.2. Mechanisms of Transition from Infantile to Adult Vocalizations

Repeated exposure to natural vocalizations has further influence on the developing cortex, promoting categorical acoustic perception. Categorical perception depends on the development of additional neurons responding selectively to complex sounds of entire vocalizations and fewer neurons responding to individual sound frequencies within the calls [303]. This mechanism facilitates recognition of species-specific vocalization types from an early age. Even if the yet undeveloped brain cannot “understand” the semiotic content of the vocalization, the statistical property of incoming sensory signals (i.e., vocalizations repeated most frequently that are likely biologically relevant) will preferentially create their categorical representation and then recognition [302].

The parallel development of the brain, and particularly the limbic system, is needed to develop control of behavioral responses and enable utilizing the categorical information formed in the auditory cortex and its association with behavioral situations. When the limbic system matures, rats begin to emit a repertoire of species-typical adult ultrasonic vocalizations and they abandon the juvenile isolation calls. Since the pup isolation calls represent aversive vocalizations, the natural extension of these calls (with negative valence) after weaning are mature, constant-frequency 22 kHz calls. Maintaining constant frequency within the call requires some regulatory skills that young pups do not have. These skills of keeping the frequency flat develop gradually from Postnatal Day 7. Maturation of oligodendrocytes and the beginning of the intensive myelination process in the brain occurs from Postnatal Day 7 [304]. At the same time, the duration of calls gradually increases from Day 7.

**Figure 2:**
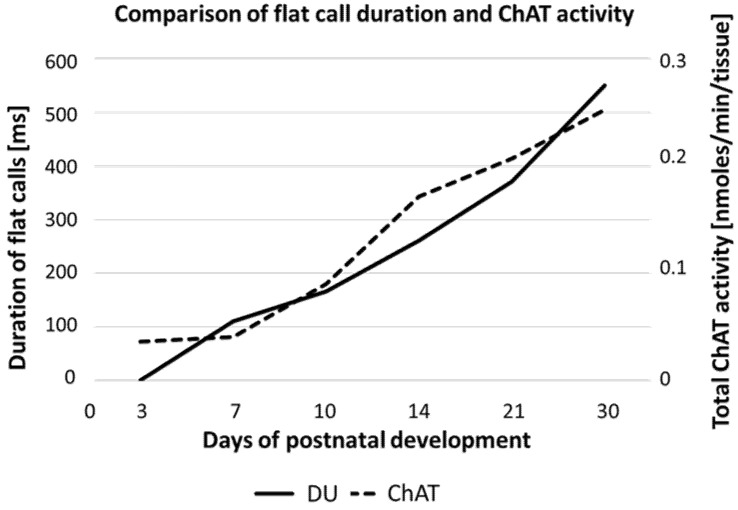
Comparison of developmental changes of duration of selected flat call (solid line) that were emitted by 3–30-day old Long–Evans rat infants with the developmental increase in the activity of choline acetyltransferase (ChAT) (dashed line) in the laterodorsal tegmental nucleus of Sprague Dawley rat pup brains. The pups’ flat calls that had peak sound frequency between 20 and 35 kHz and call duration ≥ 100 ms were selected from all emitted vocalizations in response to an air puff. There were almost no such vocalizations below the postnatal age 7, and then, 3 to 5 such flat calls were collected per day of development. Data for each point were usually collected from different pups because of the low number of emitted flat calls. The calls were measured by QML S-200 bat detector and the duration of calls were measured sonographically. The bioacoustic data are a fragment of an unpublished study that was partially reported as an abstract [305]. The measurement of ChAT activity was taken from the study by Ninomiya et al. (2001) [306]. Because the data have variable n-vales and were collected from different rat strains, the error bars were omitted. The graph shows parallel trajectories of developmental changes. Abbreviations: DU—duration of flat calls, ChAT—activity of choline acetyltransferase in the laterodorsal tegmental nucleus.

We studied in our laboratory the development of only flat calls selected from the repertoire of infantile and later juvenile vocalizations over the first month of life (Figure 2).

During the first 17 postnatal days, such calls are very rare and short [34] and they gradually appear in older rats. The constant-frequency 22 kHz calls of juvenile and adult rats are initiated by the activity of the ascending cholinergic system from the laterodorsal tegmental nucleus. This ascending cholinergic system develops poorly during the first week of postnatal development, and then rapidly accelerates over the next 7 days (Postnatal Days 7–14) and continues until weaning, which is paralleled by the increase in the laterodorsal tegmental nucleus volume [306,307]. The capability of prolonging the infantile flat calls and reaching adult 22 kHz calls is paralleled by the increase in the activity of the choline acetyltransferase, the enzyme synthetizing acetylcholine, in the laterodorsal tegmental nucleus (Figure 2) and by maturation of the respiratory system. Vocalizations that fulfilled the criterion for 22 kHz alarm calls appeared not sooner than at postnatal Day 16, although they were still relatively short.

The relationship between the growing cholinergic innervation and cholinergic initiation of early flat calls appears late, probably close to weaning. During the second week of postnatal development, the cholinergic innervation is not yet finished. Thus, the systemic application of cholinergic muscarinic agonist, oxotremorine, between Postnatal Days 10 and 17 did not potentiate the pups’ vocalizations but instead, inhibited them and it was a central effect [308]. In another study, adult rats, juveniles, and infants were subjected to standard foot shock. The rats showed the emission of different classes of ultrasonic calls to the same aversive stimulus (foot shock) [309]. While adults emitted typical 22 kHz vocalizations, juveniles emitted similar 30 kHz calls, but the infants responded with many calls grouped in two classes of calls (1) with an average main frequency of 40 kHz calls and a 300 ms call duration, and (2) an average frequency of 66 kHz calls of about 20 ms duration. Thus, the development of the brain was not equally prepared at a younger age for species-typical adult signaling. The cholinergic functions are fully developed not sooner than between Postnatal Days 20 and 25. Pilocarpine, a cholinomimetic drug, decreased amphetamine-induced psychomotor activation in 20–25 day-old rats but not in younger rats [310].

### 3.5. Interpretation of Rat 22 kHz Vocalizations

#### 3.5.1. Emission of 22 kHz Calls as Expression of Anxiety

Emission of long 22 kHz vocalizations by adult rats have been unequivocally associated with aversive situations (see Table 1 and Section 2.4 and Section 2.7, above). Regardless of the behavioral situation and function, the common denominator of these emissions is emotional arousal, reflecting a state of anxiety (not fear) [18,211,311,312,313,314,315].

It might be beneficial to provide a brief explanation of the difference between anxiety and fear that is frequently confused in publications [316]. Each of these states has different neurochemical setting and different behavioral outcome. In brief, anxiety is defined as a lasting negative state to an unknown and/or unpredictable threat, whereas fear is an acute response to a known and perceived external threat. The difference between the state of anxiety and fear is explained in the best way by a “predatory imminence continuum” that is defined by the physical (spatial and temporal) distance from rats to the approaching predator [317]. When a predator is approaching from a certain far distance (relative safety, but the predator’s behavior cannot be predicted), or its exact location is unknown, a state of anxiety appears and it may last for a prolonged time. When the predator is too close and ready to strike and the rat is without possibility of escaping from it, the fear response is initiated and it is a short-lasting response, forcing the rat to immediate action. During the anxiety state, rats vocalize intensively with alarm 22 kHz calls. On the other hand, the fear response is either silent or audible squeals are emitted directly to the predator as a warning, and the rat is ready for “fight or flight” [173].

It has been suggested that the emission of rat 22 kHz calls represents the evolutionary vocal homolog of human crying and that 22 kHz calls and human crying both express anxiety and anhedonia [315]. The emission of these aversive vocalizations is stereotypic for the species, repetitive, and innate (for both rats and humans), so the organisms do not need to learn how to emit the crying calls. A comparative study of ultrasonic vocalizations among the main strains of laboratory male rats confirmed that species-typical, adult 22 kHz ultrasonic calls were comparable among Wistar, Long–Evans, and Sprague Dawley strains with only minor acoustic differences [36]. Results from female rats from these strains were also comparable, although many females, particularly of the Wistar strain, did not emit 22 kHz calls during the fear conditioning paradigm [318], so their sensitivity to aversive situations may be different than males.

#### 3.5.2. Emission of 22 kHz Calls in Depression and Pain

The emission of 22 kHz calls and crying vocalizations in other mammals do not directly signal depression, although they are often the secondary, comorbid result of a depressive mood. The anxiety-driven emission of calls is an outward response directed to other members of the species, while depression is a withdrawn, inward response with different characteristics and without social signaling. Thus, the emission of 22 kHz vocalizations signals anxiety and should not be regarded as a direct index of depression in rats [315]. The emission of 22 kHz, however, was used as an indirect measure of a mixed affective state after social defeat in rats with some elements of depression, but the predominance of anxiety was signaled by these calls [319].

It should also be also emphasized that ultrasonic 22 kHz vocalizations do not directly signal pain itself [320,321,322]. Although the emission of 22 kHz vocalizations was increased during chronic pain (chronic polyarthritis or repeated electrical stimuli) as compared to healthy rats and these calls were suggested to serve as evaluation of analgesic drugs [323,324], 22 kHz vocalizations express an affective component (anxiety) of ongoing or repeated painful experiences, not pain itself, and these calls were sensitive to morphine [321,325,326,327,328]. Pain stimuli can even inhibit ultrasonic calling, which led in the past to a very confusing interpretation [328]. In a recent study, it was shown that the emotional response to acute pain (single injection of formalin that, however, caused long lasting pain), with the emission of vocalization presented by the demonstrator rat, showed contagion to cage mates but not to non-cage mates, or to cage mates separated by a visual barrier [322]. Thus, the familiarity among rats and visual contact both contribute to emotional contagion conveyed by vocal expression of anxiety caused by lasting painful experiences.

#### 3.5.3. Emission of 22 kHz Vocalizations Requires Some Learning Experience

Although rats emit and recognize 22 kHz innately, some associative learning is needed to link these calls with aversive stimuli and situations [329]. The initial association happens most probably in the infancy stage of life (see Section 3.4.2, above). It has been shown that association of danger (foot shock) with the playback of 22 kHz vocalizations produced defensive responses that were better encoded and consolidated in memory than responses associated with any other ultrasonic call type or signal; these responses were resistant to extinction and were retained in memory for a longer time than other responses [329]. This associative learning depends on the perception of calls of other conspecifics but not the emitters’ own calls [330]. Despite the need for this associative learning, it was concluded that rats are predisposed (primed) to learn defensive behavior in response to alarm calls, even without learning [329].

The emission of 22 kHz alarm vocalizations is the principal alarming signal in rats, which was demonstrated by the observation of the behavior of pairs of naïve or fear-experienced rats. A naïve or fear-experienced receiver rat was observed in contact with another demonstrator rat that was fear-conditioned to foot shock. The receiver repeated 22 kHz alarm vocalizations of the demonstrator and showed a freezing response but only when the receiver was experienced with the foot shock (although not conditioned to it). Naïve rats did not repeat the alarming calls of the demonstrator. In addition to that, rats with a damaged auditory system failed to repeat the calls of the demonstrator rat, even if they were fear-experienced [331]. Thus, the emission of 22 kHz ultrasonic calls is the main vehicle for the social transmission of anxiety; however, learning is needed for the proper recognition of the danger signaled by 22 kHz alarm calls [331].

Perception and recognition of the aversive value of 22 kHz alarming calls produced by adult rats significantly enhanced the acoustic startle response (an index of the anxiety-type emotional response) of adult receiver rats but garnered weak response from these rats if the emissions of alarming 22 kHz calls originated from young rats [332]. This result may further imply that the structure or pattern of emissions of 22 kHz calls by experienced rats contain some additional signaling features that are recognized by the recipients and can initiate anxiety in them.

Recognizing the aversive 22 kHz calls and learning the association between these calls and behavioral situations is a critical process that occurs from a very early stage of life at the infancy level and is continued over the life span. This process is significantly aided by acoustic cortex plasticity, recognizing and responding to whole categories of vocalizations [302].

#### 3.5.4. Expression of Internal State of Anhedonia by 22 kHz Calls

In addition to what was described, the emission of 22 kHz vocalizations may express an anhedonic internal state caused by events other than external danger or a predator. The dysphoric state during withdrawal from drugs of abuse is accompanied by the abundant emission of 22 kHz calls (for details, see Section 2.8, above).

The general features of 22 kHz calls emitted by rats during withdrawal dysphoria are compatible with anxiety driven by the affective distress and frustration associated with drug withdrawal. The same type of “inconsolable crying” or “high-pitched crying” was observed in human pediatric patients during the withdrawal phase from their addictive behavior as one of the most common symptoms [333,334].

In rats, many of the withdrawal-induced 22 kHz calls were reported as short 22 kHz calls of 10–500 ms in duration [217,335], while most long 22 kHz vocalizations are 300–3000 ms in duration [188]. Short 22 kHz calls that are less common were initially reported in rats as calls of 20–300 ms in duration [336] but their behavioral role has not been defined. Based on the observations that very long 22 kHz calls were emitted in the predator situation or in response to an air-puff, while the short calls were observed during drug withdrawal, it may be suggested that long calls are emitted in the face of external danger while short calls are characteristic of an internal dysphoric state, irritation, and displeasure without a direct, external threat [217].

The emission of 22 kHz calls during withdrawal when the drug of abuse is not available, or during frustration caused by lack of availability and access to a receptive female, being separated from the male by a partition [157], may be interpreted as signals sent to other conspecifics, even if they might not be available or cannot help. These aversive situations and the resulting behavior have been explained as frustration-induced anxiety [337], and the anxiety is signaled to conspecifics. Such behavior of irritation, frustration, stress, and resulting anxiety might be associated with the activation of additional and supplementary brain mechanisms supporting emotional arousal, i.e., augmenting the emotional arousal when the goals cannot be reached. This conclusion is supported by human studies with a concurrent, functional magnetic resonance imaging recording, in which individuals were subjected to experimentally induced frustration [338]. The results showed increased activity of structures directly involved in performing the frustrating task (sensorimotor activation) and activity of structures involved in acute stress, such as the striatum, cingulate cortex, insula, and middle frontal gyrus. Thus, the brain activity during the frustrating situation increased its activity to possibly find a solution [338] while still remaining in a state of anxiety expressed vocally.

### 3.6. Interpretation of Rat 50 kHz Vocalizations

#### 3.6.1. Emission of 50 kHz Vocalizations as Expression of Hedonia

The emission of 50 kHz vocalizations has been observed predominantly in appetitive behavioral situations (see Table 1 and Section 2.2, Section 2.3, Section 2.5 and Section 2.6, above). It has been suggested that the emission of rat 50 kHz calls represents an evolutionary counterpart of human laughter [108,339]. This homology was particularly appropriate for comparing joyful childhood laughter during active play with the emission of 50 kHz calls during juvenile rats’ rough-and-tumble play [73,101,104]. The emission of 50 kHz vocalizations expresses a positive or hedonic emotional state that may be termed hedonia (a state of pleasure, from Greek *hedone*—pleasure), a pleasurable (joyful) state within physiological limits. It should be distinguished from the obsolete and unclear meaning of this word as a pathological “abnormal cheerfulness” in human psychiatric patients [340], which was earlier called delusional amenomania [341]. In a physiological sense, hedonia is signaled by rats in most appetitive states by the emission of frequency-modulated 50 kHz calls, and particularly trill calls [342]. These calls have the same principal acoustic structure among the main rat strains (Wistar, Long–Evans, and Sprague Dawley) with only small differences [37].

Many experiments indicate that the positive emotional state expressed by the emission of 50 kHz calls contains an element of expectation and “wanting” [343]. It was, indeed, observed that the emission of 50 kHz vocalizations appeared prior to rewarding social interactions, such as in rough-and-tumble play, when seeking sexual contacts [344], or in anticipation of other incentive stimuli, such as rewarding physical activity in a running wheel [345]. Thus, hedonia should not be understood as a passive state of pleasant satisfaction (consummatory or post-consummatory state) but as an active state associated with the expectation of rewarding stimuli or the anticipation of additional rewarding stimuli. The state of hedonia is, therefore, a motorically active state, not only with the expectation of rewarding stimuli but also a state of actively looking for such stimuli, acquiring them and, at the same time, emitting honest signals to other conspecifics. This state is dopamine dependent and pharmacological activation of this system by psychostimulant agents, such as amphetamine or cocaine, always induced vigorous locomotor activity [291,346,347]. The magnitude of locomotor activity, however, is subject to individual differences, a basic level of spontaneous locomotor activity, or the intensity of the inborn response to novelty [348,349].

The direct physiological evidence for hedonia comes from self-stimulation behavior, during which rats volitionally deliver electrical stimulation to their own brains, or from place-preference behavior. Using electrical brain stimulation, all brain regions that induced emission of 50 kHz vocalizations by electrostimulation (e.g., nucleus accumbens, ventral pallidum, lateral preoptic area, lateral hypothalamus, ventral tegmental area) are also known from previous studies to support vigorous self-stimulation behavior [350]. Place-preference behavior was reported after amphetamine injections that induced emission of 50 kHz vocalizations [344], confirming its hedonic nature. Despite suggestions that 50 kHz calls might be an (anxious) indicator of negative reinforcement learning [351], a recent pharmacological study has confirmed that emission of amphetamine-induced 50 kHz vocalizations reflect a hedonic state that is resistant to anxiogenic agents and, therefore, does not reflect anxiety [352]. Moreover, rats can also learn self-injection of amphetamine directly into the shell of the nucleus accumbens, further indicating hedonic nature of this activation [353].

Emission of 50 kHz vocalizations that signal the hedonic state is perceived by receivers also as a positive and rewarding signal that can initiate a similar hedonic state in the recipients and prompt the rats to look for the cause of this behavior. This process or rapid generation of emotional arousal in the brains of receivers of vocalizations was termed ethotransmission, as a particularly fast and specific form of a broader category of behavioral transmission called emotional contagion [221]. It was even postulated that the emission of vocalizations directly targets the emotional systems of the listeners, impelling them to change their behavior [240]. Vocalization is an honest signal in rats as laughter is, in general, an honest signal in human spontaneous behavior [354]. Hence, rats showed an approach behavior to the source of the playback of 50 kHz calls as well as self-application behavior of 50 kHz calls [65,70,71,73] (for other details, see Section 2.2, above).

#### 3.6.2. Interpretation of Pharmacological Studies Inducing 50 kHz Call Emission

Results of pharmacological studies provided further support for vocal expression of the hedonic state. Application of dopaminergic drugs (cocaine, heroin, amphetamine, methamphetamine, apomorphine, quinpirole, methylphenidate) into the terminal fields of the ascending mesolimbic dopaminergic system potentiated the physiological effects of this system and induced significant emission of 50 kHz calls over the control levels [289,290,355,356,357,358]. It may be speculated that with higher doses of the drugs, this potentiation resulted in stronger hedonia than that in physiological situations, and it created a state of euphoria. This pharmacologically induced euphoric state, which has some features of mania [359], is believed to be of the same nature as the hedonic state caused by rewarding self-stimulation because all euphorigenic drugs lowered the threshold for intracranial electrical self-stimulation [360,361].

Pharmacological studies of rat 50 kHz vocalizations appeared to be a useful approach to understand the rewarding and motivational properties of drugs of abuse and the development of drug addiction in humans [362,363]. The question arose as to what value the emission of 50 kHz vocalizations expresses. Is this pure hedonic value (pleasure and liking), motivational value (wanting and motivation of incentive salience), or prediction value (expecting by learning) [343]? All these values may have separate neural mechanisms [364]. It was initially postulated that the mesolimbic dopaminergic system is mostly responsible for “wanting”, while the hedonic state (“liking”) is associated with the opioid system [365,366]. However, the emission of frequency-modulated 50 kHz calls that was induced by rewarding cues was generally found to be signaling the “liking” state with intermixed “wanting”, depending on the intensity of the motivational state of the animal [367]. Rats that attribute incentive salience to reward cues will have difficulty resisting them and were suggested to be prone to develop addiction [368].

The emission of 50 kHz vocalizations during dopamine-dependent emotional arousal (hedonia) is the activity with the signaling to conspecifics of all the aspects of positive expectation, wanting, and liking with an elevated level of locomotor activity at the same time [290,291]. Recent studies confirmed that brief optogenetic activation of the accumbens medium spiny neurons with D2 dopamine receptors increases the dopaminergic activity via effects on the ventral tegmental dopamine neurons and increases positive motivation [369] (although prolonged stimulation causes aversive effects [265]; see Section 3.2, above).

#### 3.6.3. Morphine and Emission of 50 kHz Calls

Morphine has rewarding properties but also some other unique characteristics, so it warrants a separate subsection. Acute application of morphine, a mostly μ-opioid receptor agonist, did not elevate or induce 50 kHz ultrasonic vocalizations in rats, and even had decreasing effects on the emission of these calls after withdrawal [370,371,372]. However, morphine was reported as changing the acoustic features of some subtypes of 50 kHz calls and causing a strong place-preference response [370,371]. Significant place preference was observed after intracerebral injections of synthetic peptide, μ-opioid DAMGO, directly into the ventral tegmental area, the origin site of the ascending dopaminergic mesolimbic system [350]. It was also observed that some rats emitted significant numbers of 50 kHz calls after the DAMGO injection into the ventral tegmental area (DAMGO vocalizers) while other animals did not emit 50 kHz calls (DAMGO non-vocalizers). Rats that emitted significantly more 50 kHz calls after the drug than the control (DAMGO vocalizers) showed strong place preference while the animals that did not show any increase in calling failed to show place preference [350]. It seems that opioid system is involved in the hedonic state but in a different way than the dopamine system and this is particularly observed during the long-lasting effects of drugs on the emission of 50 kHz calls [371].

The intra-accumbens injection of morphine increased social play in rats and the response was antagonized by the antagonist, naloxone [373]. Moreover, application of morphine in a certain dose-range and time after application had a decreasing effect on locomotor activity as well as anxiolytic, analgesic, and pain-alleviating effects, including a decrease in the emission of 22 kHz calls that signaled anxiety after painful stimuli [374,375,376]. These observations may indicate two reward subsystems: one early and active, dopamine-dependent subsystem; and the other with less activity and limited or no calling—an opiate-dependent subsystem [377,378] that is active during the later phase of the rewarding process.

The process of seeking and obtaining positive stimuli (preparatory phase) and experiencing pleasurable stimuli (consummatory phase) is governed by a central process that was termed hedonesthesia more than 40 years ago. It was postulated that hedonesthesia is an active and critical process for positive motivated behavior [379,380]. In the light of current knowledge, hedonesthesia is the process of appetitive emotional arousal, driven by the ascending mesolimbic dopaminergic system as well as the animal’s concomitant motor activity aiding in obtaining the positive stimuli. This process will involve many transmitters, for instance, norepinephrine at the cognitive arousal phase, dopamine at the positive emotional arousal phase, and possibly opioids at the later rewarding phase.

## 4. General Conclusions

The review summarized 22 functions of vocalizations, divided into eight groups, that play a role in rat behavior. Roughly, half of the functions are associated with negative emotional states and half with positive ones. The role of vocal communication is situation dependent and changes over a rat’s life, from a basic, life-preservation role in infants and the development of social skills in play behavior, to the resolution of social conflicts and the organization of the social group in adults as well as defense against external threats and dangers. Different types of calls in different situations and at different stages of animal life may serve as a qualitative and quantitative measure of the functioning of the animal emotional system in physiological and pathological conditions. These basic animal emotional systems are homolog to basic human affective systems—both as to neurophysiological and neurochemical mechanisms—and rat expression of emotional arousal may be used in many preclinical models.

All the rat vocal expressions, regardless of their valence, are initiated by the mechanisms of emotional arousal and are emitted in biologically important situations. The term “arousal” is used here in the same sense as in the original discovery of the ascending reticular activating system [381,382,383], i.e., as a diffuse and extensive projection systems ascending from the brainstem and directly or indirectly changing ongoing activity in the entire brain.

Emotional arousal leads to the development of one of two opposite states differing in valence: the positive, hedonic, appetitive state or the negative, anhedonic, aversive state. These two arousal states are signaled by species-specific and valence-specific ultrasonic vocalizations that are emitted to influence the behavior of other conspecifics. Pharmacological studies have proven that these vocalizations reliably reflect emotional valence and point to sets of specific receptors responsible for the appetitive or aversive state, homolog to basic limbic human brain processes.

The appetitive state is initiated by ascending mesolimbic dopaminergic projections to some forebrain structure with a hot spot in the shell of the nucleus accumbens, and releases dopamine, while the aversive state is initiated by the ascending mesolimbic cholinergic system targeting many medial diencephalic and forebrain limbic structures with hot spots in the medial hypothalamic-preoptic area and lateral septum and the release of acetylcholine. Massive release of any of these two transmitters has the capacity to rapidly change the animal’s state.

Large numbers of behavioral studies led to the conclusion that activity of the appetitive, dopaminergic system develops an active state of hedonia (pleasure in human terms) with the concurrent emission of 50 kHz vocalizations and an accompanying increase in motor activity to approach and acquire the appetitive stimuli, while the activity of the aversive, cholinergic system develops a defensive state of anxiety (displeasure) with the concurrent emission of 22 kHz calls, a decrease in motor activity and the avoidance of unpleasant stimuli.

Consistent congruence of many lines of investigation lead to the conclusion that the brain is equipped with two separate emotional arousal systems that prepare the animal for two opposite behavioral outcomes, and these systems work in parallel with the cognitive arousal. This review supports the hypothesis that all types of rat vocalizations, serving all biological functions, are driven by emotional arousal. Neural mechanisms initiating emotional arousal, positive or negative, are, therefore, common in fulfilling any of these functions.

The consistent association of 22 kHz and 50 kHz vocalizations with aversive or appetitive states, respectively, and the dual emotional arousal system makes these vocalizations particularly useful for numerous preclinical studies and models, particularly in physiological, psychological, neurological, psychiatric, and neurodevelopmental investigations. Therefore, rat ultrasonic vocalizations have been used in studies of human social psychopathologies [384], the screening of drugs for numerous conditions, particularly anxiolytic and antidepressant drugs [385,386,387], studies of schizophrenia [388], Parkinson’s disease [389], bipolar disorder [390], post-traumatic stress disorder [391], alcohol use disorders [392,393], neurodevelopmental damages [394], immunity [395], affective component of pain [229], addiction [216,335], effects of malnutrition [396] and many other disorders and diseases. The present review should help in the interpretation of the results of these and future studies.

## Figures and Tables

**Table 1:** Summary of biological and social functions of rat ultrasonic vocalizations.

Function of Vocalization	Type of Calls	Deduced Valence	Selected References
**Mother–infant communication**			
Self-preservation function	Isolation calls	Negative	[22,24]
Locating function	Isolation calls	Negative	[30,31,32,33,34,36,48]
Protective function	Isolation calls	Negative	[53,56]
**Non-agonistic adult social interactions**			
Phatic communication function	50 kHz calls	Positive	[60,61,63]
Affiliative function	50 kHz calls	Positive	[63,64,65,67,80]
Passive defensive function	22 kHz or 50 kHz	Negative or positive	[82]
Socio-coordinating function	Flat 50 kHz	Positive	[43,84]
Social buffering function	50 kHz calls	Positive	[66,80,81]
Investigative function	50 kHz calls and Short 22 kHz	Positive or negative	[100,102,103]
**Social play and teamwork**			
Ludic function	FM 50 kHz calls	Positive	[99,103,104,107,108,109]
Conative function	50 kHz calls	Positive	[48,49]
Cooperative function	50 kHz calls	Positive	[121,122,123]
**Intraspecies agonistic interactions**			
Agonistic function	22 kHz calls	Negative	[75,126,127,128,130]
Appeasement function	Long 22 kHz calls	Negative	[99,126,127,136,137]
**Reproductive behavior**			
Mating function	50 kHz or 22 kHz	Positive or Negative	[73,123,139,140,141,147]
Social detachment function	Long 22 kHz calls	Negative	[123,148,149,150,151,152,153,154,155]
**Alimentary behavior**			
Alimentary function	Flat 50 kHz calls	Positive	[164,166]
Food provisioning function	Flat 50 kHz calls	Positive	[167]
**Defense against external threat**			
Alarming function	Long 22 kHz calls	Negative	[168,169,170,171,177,188]
Warning function	Long 22 kHz calls	Negative	[191,192,193,194,195,196,197,198,199,200,201]
Security function	Long 22 kHz calls	Negative	[205]
**Expression of internal discomfort and frustration**			
Frustration expression function	Short 22 kHz calls	Negative	[148,157,213,214,215,216,217]
